# Editorial: Dietary supplements and ergogenic aids in relation to health and performance

**DOI:** 10.3389/fspor.2024.1450604

**Published:** 2024-07-03

**Authors:** Scott C. Forbes, Russ Best, Raul Dominguez

**Affiliations:** ^1^ Faculty of Education, Department of Physical Education Studies, Brandon University, Brandon, MB, Canada; ^2^ Centre for Sport Science and Human Performance, WINTEC, Hamilton, New Zealand; ^3^ Departamento de Motricidad Humana y Rendimiento Deportivo, Sevilla University, Sevilla, Spain

**Keywords:** caffeine, soccer, vitamin, nitrate, protein

**Editorial on the Research Topic** Dietary supplements and ergogenic aids in relation to health and performance

## Introduction

Dietary supplements and ergogenic aids are used to enhance exercise performance and health. When deciding to use dietary supplements or ergogenic aids, one must use an evidence-based approach. Further, it is important to examine the safety and efficacy of these dietary supplements under specific populations or to evaluate the interaction effects with other ingredients. Therefore, we created a special issue entitled “*Dietary supplements and ergogenic aids in relation to health and performance*”, which attracted several global experts in exercise nutrition. Specifically, the special issue attracted 6 high quality original manuscripts. In this editorial, we will briefly highlight key points of each manuscript.

The first study by Maestre-Hernandez et al. examined the influence of a high dose of beta-alanine supplementation (15 g/day using a sustained-release formulation) over 30 days on blood biomarkers and self-report paresthesia, a common side effect from the supplement. Interestingly, there were some small alterations in triglycerides, LDL-cholesterol, and urea nitrogen. In addition, the paresthesia appeared to be prevalent but was minor. Further, research may be warranted to explore the impact of these blood alterations on health and performance over time.

Yildirim et al. examined the effects of two doses of caffeinated chewing gum on muscular strength, vertical jump, and ball-kicking speed in trained male soccer players. Caffeine is a well-known ergogenic aid that acts as an adenosine receptor antagonist to enhance performance, however, the optimal dose when provided as a chewing gum is unknown. There were greater improvements in quadriceps strength in the 200 mg dose compared to 100 mg dose or placebo, however, no other performance metrics were improved with either dose compared to placebo.

Farra examined the ergogenic potential of a commercially available drink that contained branched chain amino acids and vitamin B6 on repeated sprint performance (5 × 1 km sprints interspersed with 2 min of active recovery) on a cycle ergometer in experienced cyclists. Power output, heart rate, ratings of perceived exertion, blood lactate and glucose, and cognition were assessed. The BCAA and vitamin B6 drink did not enhance any outcome variable, except for a small increase in post exercise blood glucose. The implications of the increase in blood glucose are unknown and may either indicate a reduction in glucose utilization or a rise in gluconeogenesis.

Nitrate supplementation is becoming more popular with a growing body of evidence demonstrating some potential to enhance exercise performance. Tan et al. extended this body of research by examining the interaction of nitrate rich beetroot juice with and without pomegranate powder on neuromuscular performance. Recreationally active males (*N* = 15) completed a randomized double-blind cross over study under three conditions: nitrate depleted beetroot juice, nitrate rich beetroot juice, or nitrate rich beetroot juice with pomegranate powder. The results revealed that beetroot juice has a small but beneficial effect, however, interestingly, pomegranate powder may compromise some of the benefits of beetroot juice.

Another study exploring the interaction of supplements was conducted by Yun et al. They found that 30 days of Rhodiola rosea followed by an acute dose of caffeine resulted in a greater increase in endurance performance and muscular power in both animal and human models compared to either supplement individually. In addition, several metabolic and markers were elevated which provides a glimpse into potential mechanisms. Again, these studies are critical to understand the interaction of different supplements.

The final study included in the special issue examined pre-sleep protein supplementation on performance, body composition and recovery in British Army recruits (Chapman et al.). This was a large study that randomized participants (99 men and 23 women) to either a control, carbohydrate placebo, a moderate dose of protein (20 g) or high dose of protein (60 g) pre sleep. Overall, the protein groups ingested more protein per day than control or placebo, however there was no effect on any performance outcome, body composition, or recovery measure. The authors noted that there was a high degree of inter-participant variability suggesting an individualize approach may be needed.

## Conclusion

This special issue included several high-quality original articles that highlights both benefits and null effects of supplements in certain conditions. Further, two of the studies explored interaction effects which are important to note when providing evidence-based recommendations.

